# Assessment of Body Condition in Long-Distance Sled Dogs: Validation of the Body Condition Score and Its Association with Ultrasonographic, Plicometric, and Anthropometric Measurements †

**DOI:** 10.3390/vetsci12080766

**Published:** 2025-08-16

**Authors:** Sergio Maffi, Alice Bonometti, Chiara Chiaffredo, Andrea Galimberti, Chiara Barletta, Katia Morselli, Laura Menchetti, Alda Quattrone

**Affiliations:** 1 Clinica Veterinaria Maffi, 25036 Palazzolo sull’Oglio, Italy; info@clinicaveterinariamaffi.eu (S.M.); segreteria@clinicaveterinariamaffi.eu (K.M.); 2 Clinica Veterinaria La Quercia, 25039 Travagliato, Italy; clinicaveterinarialaquercia@hotmail.com; 3 Clinica Veterinaria Città di Torino, 10135 Torino, Italy; chiarachiaffredo@gmail.com; 4 Clinica Veterinaria Caravaggio, 24043 Caravaggio, Italy; info@clinicaveterinariacaravaggio.com; 5 Clinica Veterinaria Fit Pet, 24041 Brembate, Italy; cdafitpet@gmail.com; 6 School of Biosciences and Veterinary Medicine, University of Camerino, Via Circonvallazione 93/95, 62024 Matelica, Italy; 7 Department of Veterinary Medicine and Animal Sciences, University of Milan, Via dell’Università 6, 26900 Lodi, Italy; alda.quattrone@unimi.it

**Keywords:** athletic dogs, working dogs, racing dogs, subcutaneous fat thickness, physical fitness, welfare and performance

## Abstract

This study aimed to validate the 9-point body condition score (BCS) system in sled dogs by assessing its reliability and by comparing it with objective measures including real-time ultrasonography, plicometry, and anthropometry. Twenty-seven Siberian Huskies (11 females, 16 males) from three sled dog teams were assessed for BCS by three trained veterinarians and their respective mushers. Intra-observer reliability was substantial (Krippendorff’s α = 0.734), while agreement between expert raters (Kα = 0.580) and between the expert rater and mushers (Kα = 0.691) was moderate, with mushers tending to overestimate the BCS of their own dogs (median difference = −0.5). BCS showed positive correlations with body mass index (BMI) and subcutaneous fat at the chest and flank via plicometry (for all: *p* < 0.05). Ultrasonography showed weak correlations with BCS, likely due to the different anatomical layers evaluated and the distinctively high muscle-to-fat ratio typical of sled dogs. Both univariate and multivariate analyses revealed sex- and neutering-related differences in body composition, with males generally presenting larger skeletal dimensions and neutering influencing patterns of fat distribution. These findings support the reliability and field applicability of the BCS system when used by trained evaluators, highlighting the importance of considering sex and anatomical site when assessing body condition in athletic dogs. The 9-point BCS, combined with accessible objective tools, represents a consistent, cost-effective method for monitoring body condition in long-distance performance sled dogs.

## Simple Summary

Monitoring body condition is essential for maintaining the health and performance of sled dogs, which are elite canine athletes exposed to high physical demands. The body condition score (BCS) is a widely used, non-invasive tool for estimating fat reserves in dogs, but its subjective nature can limit consistency. This study aimed to validate the 9-point BCS system in sled dogs and examine its correlation with objective methods including real-time ultrasonography, plicometry, and anthropometric measurements. Twenty-seven Siberian Huskies involved in long-distance racing were evaluated. Results showed that BCS had good reliability and was significantly associated with several objective parameters, with plicometry demonstrating a stronger correlation than ultrasonography. In addition, sex- and neuter-related differences in fat distribution and body measurements were identified, highlighting the importance of considering sex and physiological state in body condition assessments. These findings support the use of BCS, particularly when applied by trained evaluators, as a reliable tool for monitoring sled dog body condition. The study also emphasizes the added value of integrating simple, objective techniques to improve the accuracy and practicality of body condition evaluation in field settings, ultimately supporting better health, performance, and welfare in athletic dogs.

## 1. Introduction

Sled dogs have long played a vital role in Arctic regions, historically serving as indispensable partners for transportation, hunting, and survival in snow-dominated environments [1,2]. Although their traditional functions have declined due to mechanized transportation, climate change, and evolving Indigenous lifestyles, interest in sled dogs has resurged with the growing popularity of recreational mushing and competitive racing [3,4]. This transition has transformed them from utilitarian working animals into elite canine athletes, with breeding now focused on endurance, strength, and environmental adaptability [5,6]. A wide range of breeds participate in modern races, from traditional purebreds like the Siberian Husky and Alaskan Malamute to performance-bred types such as the Alaskan Husky and Eurohound [2,7]. Despite genetic differences, these dogs share functional traits linked to athletic performance under extreme conditions [7]. Sled dog competitions vary in format and physical demands, directly influencing the selection and training of participating dogs [6]. Long-distance races extend up to 1600 km, such as the Iditarod in Alaska, or 1200 km in Norway’s Finnmarksløpet, which is the longest sled dog competition in Europe. These endurance events take place over several days, with teams of 6 to 16 dogs sustaining average speeds of 13–19 km/h. In contrast, sprint races cover shorter distances (2–20 km), with teams of 4 to 12 dogs reaching velocities of 29–40 km/h. These competitions take place under extreme environmental conditions, usually below 0 °C and occasionally as low as −40 °C, across varied snow textures from icy to powdery. Achieving success under such demanding contexts requires not only exceptional physical and metabolic performance, but also advanced psychological conditioning and close coordination with dedicated mushers [8,9].

From a clinical perspective, the assessment of body condition is essential for monitoring the health and athletic capacity of working and sporting dogs, particularly those engaged in high-intensity disciplines such as sled dog racing. Various methods are available to evaluate body condition in dogs, each differing in terms of cost, ease of use, invasiveness, and accuracy [10,11,12,13]. In veterinary practice, the most commonly used tools are body weight (BW) and body condition score (BCS) due to their simplicity and accessibility [14]. However, BW alone may be misleading, as it does not differentiate between fat, muscle, and lean tissue, and it can vary widely even among dogs of the same breed [13,15]. In contrast, BCS offers a more reliable estimate of energy reserves, reflecting the dynamic balance between caloric intake and energy expenditure [16,17]. It is a practical, non-invasive, and standardized method that combines visual inspection and manual palpation of anatomical landmarks such as the ribs, lumbar spine, pelvis, and waist. Palpation is particularly important in northern dog breeds, where dense undercoats can obscure fat and muscle distribution [18,19]. Several BCS systems are currently in use, all based on visual and manual palpation assessment but differing in scale format. The most referenced in the literature include the 9-point scale, the 5-point scale, and S.H.A.P.E. (Size, Health, And Physical Evaluation) [17]. Their application becomes especially valuable in working and sporting dogs, where maintaining optimal body condition directly impacts performance and welfare. In the context of competitive sports, accurate and consistent BCS assessment is critical for determining a dog’s readiness for competition, guiding nutritional strategies, and training protocols. The use of standardized and validated scoring systems, most commonly the 9-point scale [20], is especially important during pre-race veterinary examinations and ongoing monitoring at veterinary checkpoints throughout long-distance races. Routine application of these systems enhances consistency across events, facilitates communication among mushers, veterinarians, and race officials, and ultimately promotes both animal welfare and optimal performance. Moreover, maintaining an optimal BCS is essential, as both excessive leanness and obesity can negatively affect thermoregulation, immune function, and physical performance, increasing the risk of injury, fatigue, and disease [16]. When dietary energy intake is insufficient to meet the elevated demands of intense exercise [21], the resulting negative energy balance leads to the mobilization of body fat reserves, and eventually, the catabolism of muscle tissue, which undermines endurance and compromises overall health and athletic performances [22].

Despite its widespread use for assessing adiposity in dogs, BCS has several notable limitations, the most significant being its inherent subjectivity. Even among experienced veterinarians, BCS evaluations can differ for the same animal, and discrepancies are further amplified among non-professionals (e.g., dog owners or mushers), who frequently underestimate or overestimate body condition compared with objective standards [23]. Additionally, while BCS demonstrates reasonable reproducibility, it lacks the sensitivity to detect subtle changes in body composition over time [10]. Advanced imaging techniques, such as dual-energy X-ray absorptiometry (DEXA), computed tomography, magnetic resonance imaging, and bioelectrical impedance analysis, have been developed to provide the precise quantification of body composition [19]. Nevertheless, DEXA is impractical for field use in sled dog research due to the size of the equipment, the need for sedation or immobilization, and the associated operational costs. Transporting athletic dogs to specialized facilities also poses logistical and welfare challenges, particularly in remote environments. As a result, both field-based and clinical studies increasingly rely on more practical alternatives such as ultrasonography, radiography, and skinfold thickness measurements with plicometry [19,24,25]. While less precise than DEXA, these methods offer greater objectivity than BCS and have shown encouraging results [11,12,24,26]. As interest grows in simple, cost-effective tools for estimating body fat in both clinical and sporting contexts, identifying the most appropriate method for sled dogs becomes essential. Validating BCS against objective techniques could enhance its accuracy and strengthen its role in monitoring canine health and performance.

In this context, we hypothesized that the BCS system would demonstrate acceptable reliability in sled dogs and show positive correlations with anthropometric measurements and subcutaneous fat thickness obtained via objective methods such as real-time ultrasonography and plicometry across multiple anatomical sites. Accordingly, the aim of the present study was to validate the BCS system in sled dogs by evaluating its inter- and intra-observer reliability and comparing it with objective measures including a range of anthropometric parameters and subcutaneous fat thickness assessed with ultrasonography and plicometry.

## 2. Materials and Methods

### 2.1. Dog Population

The study involved twenty-seven adult purebred Siberian Huskies from three Italian sled dog teams including 11 females (6 spayed, 54.5%) and 16 males (4 neutered, 25.0%). The dogs ranged in age from 1 to 12 years, with a mean age of 5 years. All dogs were at the beginning of their training season in preparation for long-distance sled dog competitions. They received routine veterinary care, were up to date with vaccinations and deworming, and were considered clinically healthy with no underlying medical condition. All parameters were evaluated on the same day for all dogs under standardized conditions. The dogs were fed a commercial kibble diet that was adequate to meet their nutritional and energy requirements [27].

### 2.2. Data Collection

#### 2.2.1. Body Weight and Body Condition Score

Body weight (kg) was measured for all dogs using a digital platform scale (KRUUSE PS250, Langeskov, Denmark).

**Figure 1:**
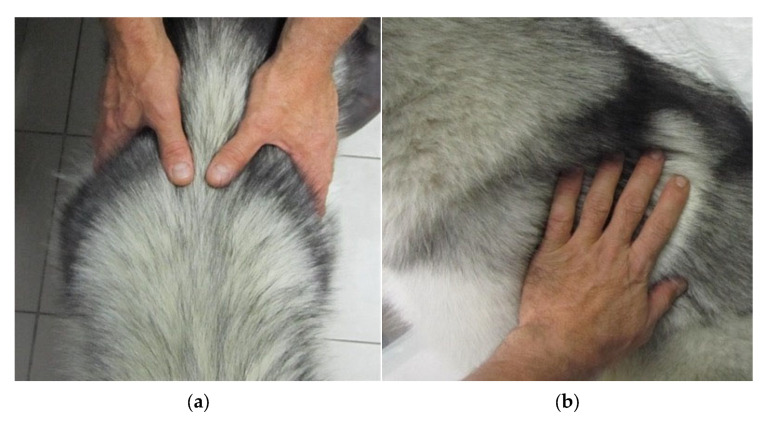
Assessment of body condition score (BCS) through manual palpation at the level of the waist (**a**) and ribs (**b**).

Each dog was assessed for body condition score (BCS) using the 9-point numerical scale proposed by Laflamme [16] and recommended by the International Sled Dog Veterinary Medical Association (ISDVMA) [28]. The BCS evaluations were conducted through visual inspection and palpation, focusing on fat and muscular coverage over key anatomical landmarks, including the ribs, scapular spine, lumbar vertebrae, pelvic bones, sternum, sacrum, all bony prominences as well as the waistline and abdominal tuck, in accordance with ISDVMA and WSAVA guidelines [29,30] (Figure 1a,b). A double-blind procedure was employed to minimize classification bias and enhance reliability involving six independent evaluators, who assessed each dog individually without exchanging information. Specifically, three veterinarians with varying levels of experience in BCS evaluation assessed all dogs. The most experienced veterinarian, with 17 years of work with sled dogs, was designated as the main evaluator, while the other two had 10 and 5 years of experience, respectively. Meanwhile, three mushers, without prior formal training in BCS assessment, evaluated only the dogs from their respective teams. The mushers were provided with visual reference charts and explanatory materials to guide their assessments. Specifically, the main evaluator conducted a 45-min training session, including practical examples, and ensured that all mushers reached agreement with the reference standards before proceeding with the evaluations.

According to the ISDVMA Body Condition Score Guidelines, the 9-point BCS scale categorizes canine body condition as follows:

- Scores 1–2 (Too thin): Indicate a severe or pronounced energy deficit. Dogs in this condition are considered too lean to safely start or continue a race.
- Score 3 (Borderline): Reflects a marginal body condition; dogs require close monitoring to ensure adequate energy intake and avoid further weight loss
- Scores 4–5 (Ideal weight): Represent the optimal condition for most sled dogs, ensuring sufficient energy reserves while avoiding excess body fat.
- Score 6 (Slightly over ideal): Still within the ideal range and considered a favorable starting condition for long-distance races in cold environments, as slightly increased energy stores can be advantageous.
- Scores 7–8 (Overweight): Slightly above the ideal range for racing dogs, this score is still acceptable for the start of endurance events in cold climates. However, it is not desirable for sprint racing or competitions in warmer temperatures.
- Scores 8–9 (Obese): Indicative of excessive body fat and generally considered unsuitable for competitive racing.

#### 2.2.2. Anthropometric Measurements

**Figure 2:**
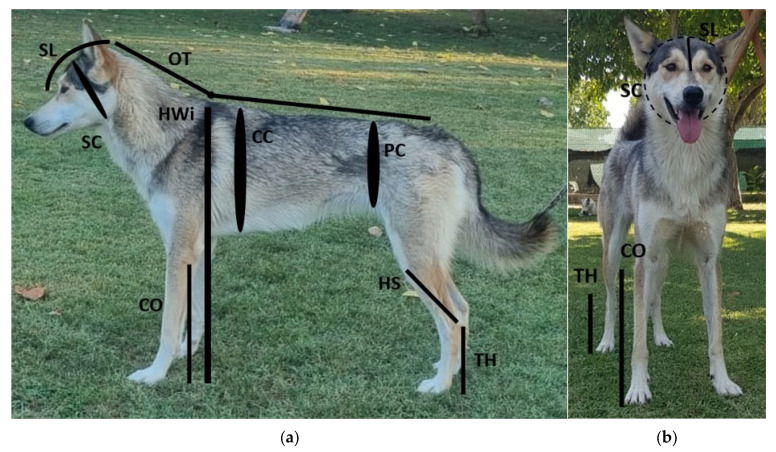
Representation of the anatomical sites and anthropometric measurements collected, shown from the lateral (**a**) and frontal (**b**) views. SL = skull length, SC = skull circumference, OT = occipital-to-tail length, Hwi = height at withers, CO = carpal pad–olecranon distance, CC = chest circumference, PC = pelvic circumference, HS = hock to stifle length, TH = tarsal pad–heel distance. The subject is Icebell’s Peppa, a Siberian Husky from Icebell’s Siberian Husky kennel. Images courtesy of Arianna Paci.

Anthropometric measurements (in cm) were obtained using a metal measuring stick (Körmaß Metall, Schweikert Dog Equipment, Bürstadt, Germany) and a retractable flexible body measuring tape (model EAM-400, Elite Medical Instruments Inc., Fullerton, CA, USA) following established methodologies [15,25,31]. All measurements were taken with the dogs in a standing position, looking straight ahead, and maintaining a natural head carriage (Figure 2).

The following anthropometric measures were obtained:

- Height at withers (HWi): Vertical distance from the ground to the dorsal limit of the scapular region.
- Chest girth (CC): Circumference of the chest measured immediately caudal to the scapula, at the widest part of the ribcage.
- Pelvic circumference (PC): Measured immediately cranial to the inguinal region around the level of the flank.
- Hock to stifle length (HS): Distance from the most caudal aspect of the tuber calcanei (hock) to the midpoint of the patellar ligament (stifle joint).
- Occipital-to-tail length (OT): Length from the occipital protuberance to the base of the tail.
- Skull circumference (SC): Circumference measured around the widest part of the skull.
- Skull length (SL): Distance from the occipital protuberance to the medial canthus of the eye.
- Tarsal pad–heel distance (TH): Distance from the proximal aspect of the tarsal pad to the most caudal point of the calcaneal tuberosity.
- Carpal pad–olecranon distance (CO): Distance from the proximal aspect of the carpal pad to the proximal tip of the olecranon.

The anthropometric measures were used to calculate the body mass index (BMI) and body fat percentage (BF%) based on the following equations [11,15]:

Body mass index (BMI) = BW_kg_/(HWi_cm_ × OT_cm_) (1)

Body fat percentage (%BF):(2)

Male body fat (%) = −1.4 (HS_cm_) + 0.77 (PC_cm_) + 4

Female body fat (%) = −1.7 (HS_cm_) + 0.93 (PC_cm_) + 5

Either gender body fat (%) = [−0.0034 (HS^2^) + 0.0027 (PC^2^) − 1.9]/BW

#### 2.2.3. Subcutaneous Fat Thickness (SFT) Measurement

SFT was measured in mm at multiple anatomical sites using both real-time ultrasonography and plicometry.

**Figure 3:**
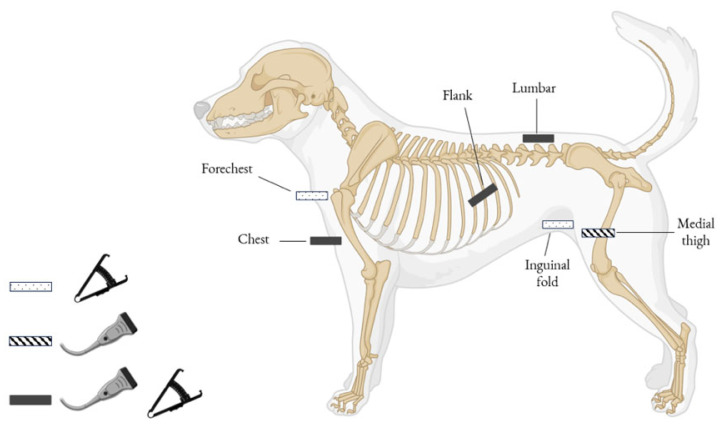
Schematic representation of the anatomical sites used in the study to assess subcutaneous fat thickness in sled dog using real-time ultrasonography and plicometry.

**Figure 4:**
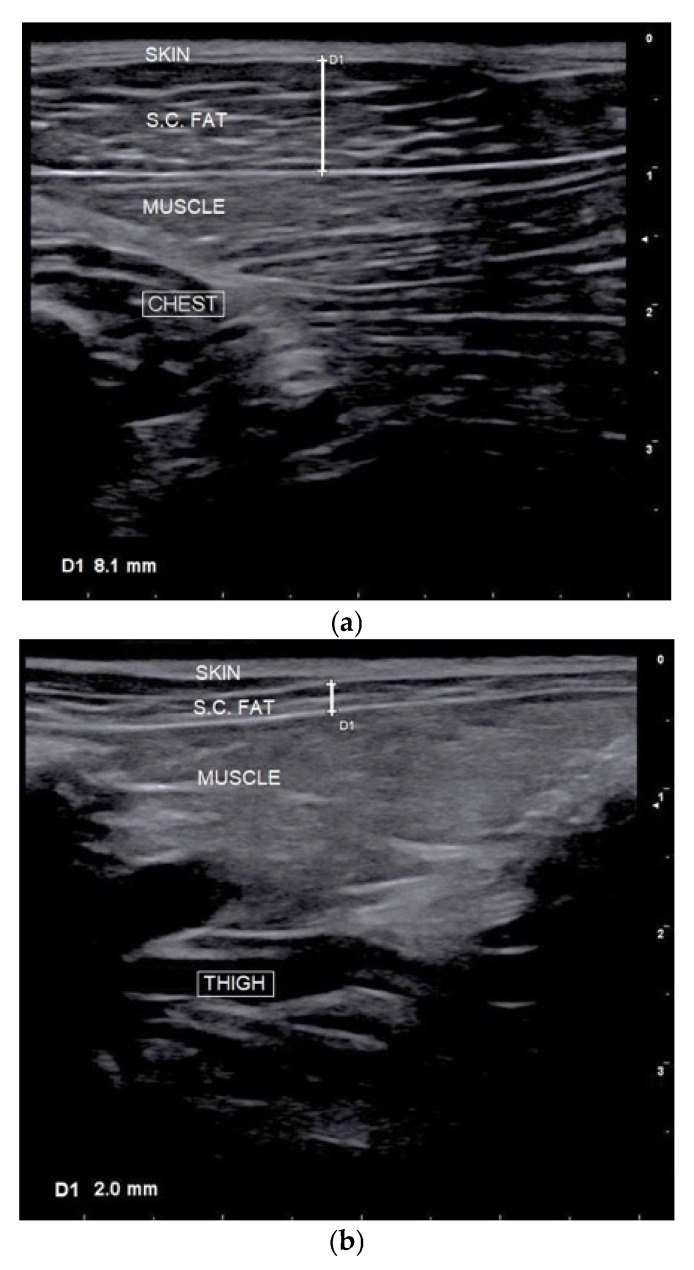

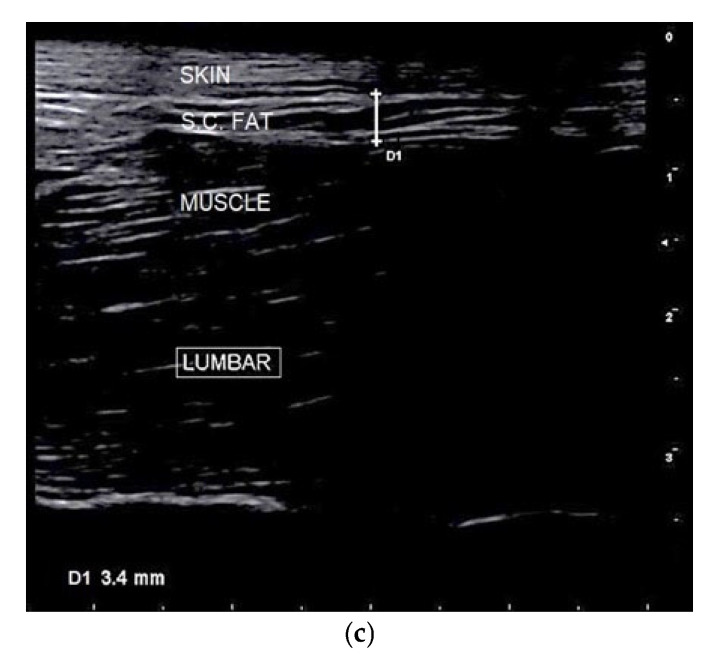
Real-time ultrasonography assessment of subcutaneous fat thickness (in mm) in sled dogs. Representative images from the anatomical sites sampled: (**a**) chest, (**b**) medial thigh, and (**c**) lumbar. In each image, measurements of subcutaneous fat thickness (SFT) were taken three times to estimate the mean value.

Real-time ultrasonography was conducted using a MyLab™30GoldVET ultrasound device (Esaote S.p.A., Genova, Italy) equipped with a 12 MHz linear probe. Image acquisition followed previously validated protocols in canine studies [11,12,24] and was performed at four predefined anatomical sites: chest, flank, thigh, and lumbar region [11,12] (Figure 3 and Figure 4). All measurements were taken with the dogs in a standing position, without the use of sedation, anesthesia, or hair clipping. Ultrasound gel and fluid (alcohol) were applied as a coupling medium to optimize acoustic contact. To ensure consistency and reduce inter-operator variability, a single experienced operator conducted all assessments. At each site, three measurements were recorded, and the mean value was used for subsequent analysis. In parallel, SFT was assessed via plicometry using a skinfold caliper (BOZEERA^®^ Body-Fat Caliper PRO, Nijmegen, The Netherlands) at five anatomical sites: chest, forechest, flank, lumbar, and inguinal fold. While three of these sites (chest, flank, and lumbar) overlapped with the ultrasound examination, the forechest and inguinal fold were included exclusively in the plicometric evaluation to broaden the assessment of subcutaneous fat distribution (Figure 3). These anatomical locations were selected based on their documented relevance for evaluating body fat in dogs, as reported in previous studies [11,12,17,32]. The measurements were performed by two veterinarians with different levels of experience in plicometry (one highly experienced and the other less experienced).

The specific anatomical sites (Figure 3) and orientations for each measurement were as follows:

(1) Chest (ultrasound and plicometry): The probe or caliper was positioned transversely to the manubrium sterni, slightly left of the midline, over the cleidocephalicus muscle at the cranial thoracic inlet (Figure 4a).

(2) Forechest (plicometry): STF was measured at the ventral cervical-thoracic junction, along the midline, at the level of the manubrium but more dorsally than the chest site. The site corresponded to the transition zone between the caudal cervical region and the cranial thorax, approximately midway between the two scapulohumeral joints.

(3) Flank (ultrasound and plicometry): On the left abdominal wall, the probe or caliper was positioned transversely over the 9th intercostal space, just dorsal to the costochondral junction, over the external oblique abdominal muscle;

(4) Medial thigh (ultrasound): Measurements were taken on the medial aspect of the right pelvic limb, with the probe positioned transversely to the femur, midway along an imaginary diagonal line connecting the ischiatic tuberosity to the tibial tuberosity, between the gracilis and semimembranosus muscles (Figure 4b);

(5) Lumbar (ultrasound and plicometry): The probe was aligned longitudinally, parallel to the spinous processes of the 3rd and 5th lumbar vertebrae, placed left of the dorsal midline (Figure 4c).

(6) Inguinal fold (plicometry): The skinfold was measured at the natural fold extending from the medial aspect of the femorotibial (stifle) joint to the flank, near the inguinal region and adjacent to the abdominal wall. This site corresponds to the natural cutaneous fold formed between the medial surface of the thigh and the ventrolateral abdominal wall, close to the insertion area of the abdominal musculature.

### 2.3. Data Analysis

Descriptive statistics were used to present the data as means, standard deviations, and ranges. Diagnostic graphs, the Kolmogorov–Smirnov, and Levene’s tests were used to check the assumptions of normality and homoscedasticity as well as the presence of outliers. Hock-to-stifle length (HS), skull length (SL), and tarsal pad–heel distance (TH), chest and flank measurements taken with ultrasonography as well as chest measurements taken with plicometry were log-transformed to improve their distribution. To evaluate the effects of sex and neuter status on the body condition measurements, a two-way analysis of variance (ANOVA) was conducted. Sex (male/female) and neuter status (intact/neutered) were included as fixed factors in the model. The interaction between sex and neutering was also included to assess potential combined effects on body condition. Since no transformation improved the distribution of height at withers (HWi), this variable was analyzed using non-parametric methods. In particular, independent Mann–Whitney U tests were used to assess the separate effects of sex and neuter status on the body condition measurements. Additionally, a Kruskal–Wallis test was performed on dogs grouped by the combination of sex and neuter status (i.e., intact females, spayed females, intact males, and neutered males).

The associations between sex and neuter status and BCS (the average of the two measurements taken by the main evaluator, rounded up to the whole) were evaluated by the eta coefficient. Eta is a measure of nominal-by-interval association that ranges from 0 to 1, with 0 indicating no association between the variables, and values close to 1 indicating a high degree of association [33]. In particular, as a measure of effect size, eta was interpreted as a small association if it was <0.3, medium if 0.3 ≤ eta < 0.5, and large if the eta was ≥0.5 [34]. Moreover, a *z*-test was used to compare column proportions.

Table 1 summarizes the statistical methods used to test different aspects of the validation of the BCS in sled dogs including its reliability and validity [35,36,37].

Generalized linear model results also express the association between BCS and real-time ultrasonography and plicometry.

Moreover, a multivariate approach was used to find a dimension describing the body condition of the dogs. First, the variables that demonstrated statistical significance in the GLM models at a significance level of *p* < 0.1 (i.e., chest girth and BMI as well as chest, flank, forechest, lumbar, and inguinal fold evaluated using plicometry) were included in principal component analyses (PCAs) to construct equations with the objective measurements that describe the BCS. Four separate PCAs were performed, stratified by sex and neuter status (i.e., intact females, spayed females, intact males, and neutered males). For each PCA, one principal component (PC) with an eigenvalue greater than 1 was extracted, representing the underlying body condition dimension within each group. The first component is, indeed, the linear combination of the variables that accounts for the maximum amount of variance. Only factor loadings with an absolute value greater than 0.4 were interpreted [40,41]. The Kaiser–Meyer–Olkin (KMO) test was used to check the sampling adequacy of the PCAs.

Finally, intra- and inter-observer agreement (n = 2) for the plicometric measurements was assessed using intraclass correlation coefficients (ICCs), appropriate for continuous variables. ICCs were calculated based on a two-way ANOVA model with absolute agreement. Single-measure ICCs were used to evaluate intra-observer reliability, while average-measure ICCs were used to assess inter-observer reliability. ICC values were interpreted according to the same criteria applied to the Kα coefficient [34].

The statistical analyses were performed with SPSS 25.0 software (SPSS Inc., Chicago, IL, USA), whereas the figures were generated using GraphPad version 7 (GraphPad Software, San Diego, CA, USA). A *p*-value ≤ 0.05 was considered statistically significant.

## 3. Results

### 3.1. Anthropometric Measurements: Descriptive and Sexual Dimorphism

Table 2 and Table 3 present the descriptive statistics for the evaluated measurements and the observed sex-and neutering-related differences. Males exhibited significantly higher values than females for several variables including height at withers (HWi, *p* = 0.001), chest circumference (CC, *p* = 0.034), hock-to-stifle length (HS, *p* = 0.013), and carpal pad–olecranon distance (CO, *p* = 0.002). For HWi, the Kruskal–Wallis test also indicated significant differences among the four groups defined by sex and neuter status (*p* = 0.009); however, pairwise comparisons revealed significant differences only between males and females (*p* < 0.05).

A trend toward significance (*p* < 0.10), with males showing higher values than females, was also observed for body weight (BW), occipital to tail-base length (OT), tarsal pad to heel distance (TH), and subcutaneous fat thickness at the lumbar level measured via real-time ultrasonography.

Interestingly, some plicometric measurements showed the opposite pattern, with females exhibiting significantly higher values than males for forechest (*p* = 0.028) and inguinal fold (*p* = 0.011) subcutaneous fat thickness.

Regarding the effect of neutering, a borderline significant reduction in chest subcutaneous fat thickness assessed via ultrasonography was found (*p* = 0.050), with a tendency for this measure to be greater in females than in males (*p* < 0.10). Finally, flank and lumbar plicometric measurements showed a significant interaction between sex and neutering status: spayed females had significantly lower values compared with intact females (*p* < 0.05), while no neutering-related differences were detected among males.

As for BCS (Table 3), a strong association was found with sex/neutering status (eta = 0.701). Specifically, the highest proportion of dogs with lower BCS values (i.e., score 4) were intact males. In contrast, among dogs with higher BCS values, the majority of those scoring 6 were neutered males, while the highest proportion of dogs with the highest BCS (i.e., score 7) were spayed females (*p* < 0.05; Table 3).

### 3.2. BCS Validation: Intra and Inter-Observer Agreement

Intra-observer agreement for the BCS values was substantial (Kα = 0.734; bootstrap confidence interval 95% = 0.448–0.882) while the agreement between expert raters (Kα = 0.580; bootstrap confidence interval 95% = 0.275–0.766) as well as between the expert rater and the musher was moderate (n = 3; Kα = 0.691; bootstrap confidence interval 95% = 0.339–0.845). The median difference between the BCS assigned by the main rater and that assigned by the other veterinarian evaluators was 0.25 (range: −0.5 to 2), while the median difference between the BCS assigned by the main rater and that assigned by the musher was −0.5 (range: −2 to 1), indicating a tendency for mushers to overestimate their dogs’ BCS.

Regarding the intra-observer agreement of the measurements obtained using plicometry, all parameters demonstrated substantial reliability (for all: ICC > 0.6, *p* < 0.001; Table 4). In contrast, the inter-observer agreement of plicometry was substantial for the chest, flank, and lumbar regions, whereas the forechest and inguinal fold showed only fair agreement (ICC < 0.40; Table 4).

### 3.3. BCS Validation: Concurrent Validity

**Figure 5:**
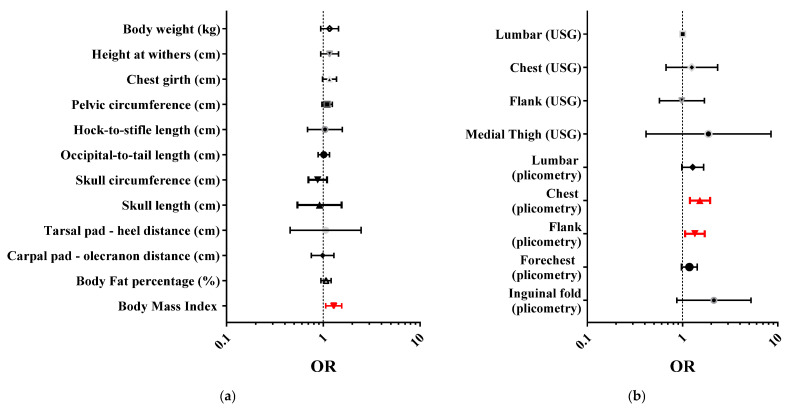
Odds ratio (OR) and 95% confidence interval for the association between the BCS and anthropometric measurements and calculated index (**a**), and between the BCS and measurements collected with real-time ultrasonography and plicometry (**b**). Red bars and symbols indicate variables with statistically significant ORs (*p* < 0.005).

Figure 5 and Table S1 show the results of GLMs indicating the strength of the association between the BCS and anthropometric measurements including the calculated indices (Panel a) and between BCS and measurements collected with real-time ultrasonography and plicometry (Panel b). A positive association was found between BCS and BMI (*p* = 0.010), chest evaluated using plicometry (*p* = 0.003), and flank evaluated using plicometry (*p* = 0.036). A trend (*p* < 0.1) was found for chest girth, forechest, inguinal fold, and lumbar measurements taken with plicometry.

### 3.4. Multivariate Analyses Defining the Body Condition of Intact and Neutered Male and Female Dogs

To explore potential group-specific patterns, separate PCAs were performed for intact and spayed females and intact and neutered males using the same set of variables.

The highest proportion of variance explained by the PCAs was observed in neutered dogs (i.e., 91.0% in spayed females and 83.5% in neutered males). However, a substantial percentage of variance was also explained in intact animals, with 72.1% in intact males and 62.9% in intact females.

**Figure 6:**
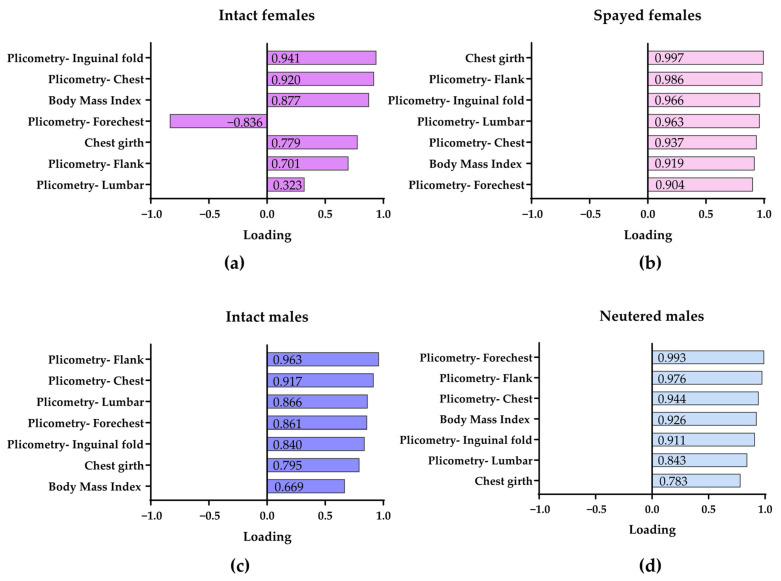
Factor loadings for intact (**a**) and spayed (**b**) females and intact (**c**) and neutered (**d**) males.

The factor loadings of the extracted PCs for intact and spayed females and intact and neutered males are presented in Figure 6. The resulting loadings differed across groups, suggesting distinct underlying structures in body composition. In particular, females tended to show higher loadings than males for inguinal fold thickness and chest girth, with particularly high values observed in spayed females. Interestingly, unlike the other groups, intact females exhibited a negative loading for forechest skinfold thickness, while intact males showed a low loading for BMI. Spayed females differed from intact ones not only in having a greater chest girth, but also in showing higher loadings for flank and lumbar skinfold thickness. In neutered males, both forechest skinfold thickness and BMI loadings were higher compared with intact males.

## 4. Discussion

To our knowledge, this is the first study to validate the 9-point BCS system for long-distance sled dogs, and to compare it with objective measures of body composition. Its simplicity, reproducibility, and standardized visual charts have made it widely used for evaluating the body condition of athletic dogs, although it remains a subjective method and is susceptible to inter-observer variability. Thus, its validation is particularly important for sled dogs, whose health and performance are closely linked to optimal body condition, highlighting the need for reliable and practical assessment tools in the field.

In our study, inter-observer agreement was moderate among both veterinarians and between the most experienced evaluator and the mushers. However, mushers consistently overestimated body condition, assigning higher BCS scores than veterinarians. This contrasts with findings in other working dog populations and pets, where owners often underestimate their dogs’ BCS [23,42,43,44]. This discrepancy observed in our study may reflect the specific context of sled dogs, in which the mushers’ perceptions of their dogs’ muscularity or fitness may be shaped by emotional attachment as well as by preconceived expectations of what an athletic dog should look like. In contrast, greater experience, lack of emotional bias, and familiarity with BCS protocols are likely to contribute to more objective scoring by veterinarians. This reinforces the critical need for employing well-trained evaluators and standardizing assessment practices. In sled dog races, the importance of this training is particularly highlighted by the fact that BCS represents a key welfare parameter assessed at checkpoints to determine the dogs’ fitness to continue competing. Excessive weight loss and BCS values below the acceptable threshold can result in veterinary exclusion from the race to safeguard the dogs’ health. While some variability between evaluators was expected due to the subjective nature of BCS, the agreement level observed in our study indicates acceptable consistency and aligns with previous reports showing that evaluator interpretation can influence scoring [17,23,24,45]. Furthermore, our substantial intra-observer agreement confirms a good level of repeatability. Together, these findings support the validity of the 9-point BCS system in long-distance sled dogs, particularly when applied by trained professionals, and emphasize the importance of training and standardization to minimize subjectivity [44,46,47].

To further validate the use of the 9-point BCS system, we also analyzed its correlation with objective measures of body condition including anthropometric measurements and subcutaneous fat thickness assessed via real-time ultrasonography and plicometry. Among the anthropometric parameters, we observed a positive correlation between BCS and BMI, which aligns with previous studies showing that BMI can reflect body condition, though less precisely than direct fat measures [17,48]. While anthropometric measurements are valuable tools for objectively assessing body composition by quantifying anatomical lengths and circumferences and monitoring their changes over time [25,31], their clinical application in dogs remains limited, mainly due to factors such as breed variability in size and body conformation [13,17]. Practical limitations also include the difficulty of obtaining accurate measurements in awake animals, the time required (up to 10 min), and potential interference from coat thickness [13,17,25]. Despite these limitations, the method is considered reliable due to its low inter- and intra-rater variability, even without specialized training [25].

Interestingly, our anthropometric results revealed significant sexual dimorphism. Males exhibited greater values for key skeletal measurements, including height at the withers, hock-to-stifle length, chest circumference, and carpal pad–olecranon distance, consistent with prior research including the Siberian Husky [49,50,51]. Conversely, females displayed significantly greater subcutaneous fat thickness measured by plicometry at the forechest and inguinal fold levels. This aligns with established evidence that females of many mammalian species, including dogs, generally have higher body fat reserves due to hormonal influences, particularly estrogen, which promotes lipid deposition and modulates fat distribution patterns [52]. Regarding neuter status, spayed females showed lower flank and lumbar plicometric fat thickness compared with intact ones, likely reflecting the metabolic and endocrine changes following gonadectomy including reduced estrogen levels that can alter regional fat distribution and overall body composition [53,54]. Such differences were not found in males based on neuter status, possibly due to the lesser role of androgens in regulating fat deposition patterns [55].

The effect of neuter status was more evident when considering BCS scoring. The majority of dogs in this study fell within the ideal range for endurance sled dogs (scores 4–6). However, intact males mostly scored at the lower end (i.e., BCS = 4), while all neutered males scored at the higher end of the ideal range (i.e., BCS = 6). That said, in sled dogs, a BCS of 6 may be beneficial, particularly in cold environments where additional energy reserves are advantageous at the onset of a race. Among the few dogs with higher BCS values (score 7), all were females, and most were spayed. While a BCS of 7 is generally considered slightly overweight for racing dogs, it is acceptable as a starting condition for long-distance events in cold climates, although it is less desirable for sprint races or competitions held in warmer temperatures. These results are consistent with previous studies reporting an association between gonadectomy and increased adiposity in dogs, potentially mediated by a reduction in basal metabolic rate, decreased energy expenditure, and changes in appetite regulation [53]. Although all dogs in this study were actively training and within ideal BCS range, the tendency for higher scores to occur predominantly in spayed females highlights the importance of considering both sex and neuter status when assessing and managing body composition. Furthermore, the neuter-related differences detected using BCS support its validity as a tool for evaluating body condition in sporting dogs.

Regarding subcutaneous fat thickness, BCS correlated more strongly with plicometry than with the ultrasonographic measurements. This stronger association may be explained by the similarity of the anatomical layers assessed: plicometry measures compressed dermis and subcutaneous fat, closely matching the tactile evaluation used in BCS scoring, whereas ultrasonography differentiates tissue types and measures adipose tissue alone [11,12,24]. This higher agreement between BCS and plicometry may also reflect the relevance of the chosen anatomical sites, as fat thickness in regions like the chest and abdominal areas is known to correlate strongly with total body fat [11]. The reliability of our plicometric measurements was supported by substantial intra-observer agreement across all anatomical sites. In contrast, low inter-observer agreement was observed for certain subcutaneous fat thickness measurement sites, such as the forechest and inguinal fold, which could reflect the anatomical complexity and variability of fat distribution in these areas as well as differences in operator experience. Considering the relatively low cost, portability, and ease of use of skinfold calipers in the field, plicometry may serve as a practical complementary tool to BCS in the routine monitoring of body condition in athletic dogs.

On the other hand, the poor correlation between BCS and ultrasonographic subcutaneous fat thickness was unexpected, as previous studies have generally reported strong associations between ultrasound measurements and both the total body fat and BCS in dogs [11,12,24]. Several factors may explain this weak correlation including variation in anatomical site selection, the subjectivity of BCS scoring, and the technical sensitivity of ultrasonography to factors such as probe placement, applied pressure, and operator expertise. These challenges are amplified in lean, athletic dogs like sled dogs, where fat deposits are minimal and harder to assess accurately [56]. Additionally, the high muscle-to-fat ratio typical of these dogs may further weaken the relationship between palpation-based BCS and localized fat thickness detected with ultrasound. Importantly, our findings underscore a fundamental limitation of BCS: while it offers a general estimate of body fat reserves, it does not differentiate between adipose tissue, muscle mass, or lean body mass [18]. Therefore, for a more comprehensive body composition evaluation, it is crucial to combine BCS with more objective, region-specific methods, as relying on visual inspection and palpation alone may overlook subtle but clinically relevant changes in fat and muscle distribution [11]. This is particularly important in athletic dogs, such as sled dogs, where optimal performance depends on a precise balance between lean muscle mass and adequate energy reserves [56].

Our multivariate analysis provided further support for sex-and neutering-related differences in body composition. The higher percentage of variance explained by the PCAs in neutered animals suggests a more homogeneous pattern of body composition within these groups, likely driven by a dominant factor such as fat accumulation following neutering [48]. In contrast, the lower variance explained in intact animals may reflect greater biological variability influenced by hormonal and metabolic differences. Moreover, the observed differences in factor loadings across sex and neutering status suggest distinct patterns of body composition among the groups. In females, inguinal fold subcutaneous thickness and chest girth showed higher loadings compared with males, particularly in spayed individuals, indicating that these regions contribute more prominently to the variation in body condition. Spayed females also displayed higher loadings for flank and lumbar subcutaneous fat thickness, supporting a shift toward regional fat accumulation post-neutering. Interestingly, intact females exhibited a markedly low and negative loading for forechest skinfold thickness, suggesting that adiposity in this region may follow an independent or even opposite trend relative to the overall body condition profile in this group. This finding could reflect a more complex or heterogeneous distribution of fat in intact females. BMI had a low contribution to the body condition dimension in intact males, suggesting that it may be a less sensitive index in this group, possibly due to greater variability in lean mass. Conversely, in neutered males, both BMI and forechest skinfold thickness showed higher loadings, pointing to a more generalized and uniform pattern of fat deposition, consistent with known post-castration metabolic changes.

## 5. Conclusions

The 9-point BCS system provides good consistency and reliability, showing significant correlations with objective measures of overall body condition, while also capturing sex- and neutering-specific patterns. This supports its value, particularly for use in the performance of long-distance sled dogs. Nevertheless, accurately assigning a score that distinguishes between adipose tissue, muscle, and lean mass may be challenging, underscoring the importance of adequate evaluator training. Notably, observed sex- and neutering-specific differences in fat distribution and skeletal morphology highlight the importance of considering sex and neuter status during assessments. Given the distinct physiological demands of athletic dogs, employing multiple assessment tools allows for the more precise monitoring of fat reserves and lean muscle mass. Future research should focus on refining non-invasive, cost-effective methods for evaluating body condition in athletic dogs, with consideration for breed, sex, and physiological status to enhance field applicability and clinical decision-making.

## Figures and Tables

**Table 1:** Statistical methods used to test different aspects of the validation process for the body condition score (BCS) in sled dogs.

Type	Definition	Statistic Used	Criteria
Inter-observer reliability	Agreement between multiple expert people (n = 3) and between the main observer and the musher * Independently rating the same dog [35].	Krippendorff’s alpha (Kα; using 1000 bootstrap samples to estimate the 95% confidence interval) [36].	Krippendorff’s alpha (Kα) coefficient was interpreted as none to slight (0.01 ≤ Kα < 0.20), fair (0.21 ≤ Kα < 0.40), moderate (0.41 ≤ Kα < 0.60), substantial (0.61 ≤ Kα < 0.80), and almost perfect (Kα ≥ 0.81) agreement [36,38].
Intra-observer reliability	Agreement between ratings by the same individual (the most experienced evaluator) on multiple evaluations [35].		
Concurrent validity	The extent to which BCS scores have a stronger relationship with the criterion (gold standard) measurement [39].	Generalized linear models (GLM) with multinomial as probability distribution and cumulative logit as link function. The BCS was included as the dependent variable, and objective measurements as independent variables. The three models (one model for the measurements taken with centimeters, one for those of ultrasound and one for those taken by plicometry) evaluated the main effect of the measurements.	The average of the two assessments of the most experienced evaluator was considered the most reliable estimate of the BCS. The objective measurements taken with centimeters, ultrasound, and plicometry were used as the gold standard. The strength of the association between BCS and gold standard measures was expressed as the odds ratio (OR) with a 95% confidence interval (CI) and the *p* value [33].

* Each musher only evaluated their own dogs.

**Table 2:** Descriptive statistics stratified by sex and neuter status for anthropometric data, calculated indices, and subcutaneous fat thickness measured via real-time ultrasonography and plicometry, along with the significance of the effects (sex, neutering, and their interaction). M = mean, SE = standard error, Md = median, Min = minimum, Max = maximum. *p*-values in bold were significant at the 0.05 level.

Measurement		Sex and Neuter Status																				*p* Value		
		Female Intact					Female Spayed					Male Intact					Male Neutered					Sex	Neutering	Interaction
Technique	Parameter	M	SE	Md	Min	Max	M	SE	Md	Min	Max	M	SE	Md	Min	Max	M	SE	Md	Min	Max			
**Anthropometric measurements**	**BW (kg)**	21.5	1.2	21.8	17.3	24.4	20.6	1.4	19.0	17.8	25.5	22.3	1.0	21.6	17.8	27.9	24.5	1.4	25.9	20.2	26.0	0.087	0.618	0.217
	**Hwi ^†^ (cm)**	54.4	0.9	55.0	51.0	56.0	54.7	1.3	54.0	51.0	60.0	58.1	0.6	58.5	52.0	60.0	59.1	1.0	59.5	56.5	61.0	**0.001**	0.863	**0.009**
	**CC (cm)**	65.2	0.9	66.0	62.0	67.0	65.3	1.9	63.5	61.0	72.0	66.6	1.0	65.5	61.0	72.0	71.0	2.5	72.0	64.0	76.0	**0.034**	0.160	0.185
	**PC (cm)**	50.0	0.8	51.0	47.0	51.0	46.7	2.3	45.5	40.0	54.0	48.8	1.6	46.0	42.0	58.0	53.3	3.0	51.5	48.0	62.0	0.233	0.809	0.093
	**HS ^○^ (cm)**	20.8	0.4	21.0	20.0	22.0	20.8	0.8	20.5	18.0	24.0	22.0	0.4	22.5	20.0	24.0	23.0	0.8	23.0	21.0	25.0	**0.013**	0.472	0.437
	**OT (cm)**	75.8	1.7	76.0	70.0	79.0	73.2	1.0	73.0	70.0	76.0	76.9	1.5	77.0	69.0	88.0	79.5	3.0	81.5	71.0	84.0	0.066	0.990	0.190
	**SC ^○^ (cm)**	39.0	1.5	38.0	36.0	44.0	39.3	1.1	39.5	35.0	43.0	41.5	1.0	41.5	37.0	47.0	40.8	0.8	40.0	40.0	43.0	0.310	0.528	0.474
	**SL (cm)**	13.0	0.0	13.0	13.0	13.0	12.3	0.3	12.0	12.0	14.0	13.3	0.5	13.0	11.0	16.0	13.3	0.6	13.0	12.0	15.0	0.279	0.532	0.532
	**TH ^○^ (cm)**	14.2	0.2	14.0	14.0	15.0	14.7	0.3	14.5	14.0	16.0	15.0	0.2	15.0	14.0	16.0	15.3	0.5	15.5	14.0	16.0	0.052	0.298	0.743
	**CO (cm)**	27.2	0.4	27.0	26.0	28.0	27.0	1.2	27.5	22.0	30.0	29.3	0.6	29.5	25.0	32.0	30.8	0.9	31.5	28.0	32.0	**0.002**	0.484	0.355
**Calculated index**	**% BF**	15.9	1.2	15.4	12.2	19.4	12.0	1.9	12.7	4.3	17.1	13.1	1.9	11.3	6.1	27.5	16.3	3.2	14.0	11.5	25.7	0.750	0.876	0.141
	**% BF ^‡^**	16.1	1.0	15.8	13.0	18.4	13.0	1.8	13.6	4.8	18.6	10.8	1.4	9.8	5.5	20.7	12.8	2.4	11.7	8.3	19.5	0.150	0.758	0.177
	**BMI**	51.9	1.9	52.5	45.2	56.2	51.4	2.2	50.6	44.7	57.6	49.9	1.4	48.4	43.6	58.8	52.0	1.1	51.3	50.4	55.1	0.717	0.689	0.496
**Real-time ultrasonography (mm)**	**Chest ^○^**	3.5	0.5	3.5	2.2	4.7	2.5	0.6	2.1	1.4	5.0	2.5	0.3	2.2	1.5	5.0	2.2	0.3	2.0	1.7	2.9	0.081	**0.050**	0.148
	**Flank ^○^**	4.5	0.9	4.4	2.4	7.6	2.7	0.5	2.2	1.7	4.6	3.3	0.3	3.0	1.7	6.3	3.7	0.1	3.7	3.6	3.9	0.775	0.716	0.209
	**Medial thigh**	2.6	0.3	2.6	1.7	3.3	2.1	0.2	2.1	1.5	2.9	2.2	0.1	2.2	1.6	2.8	2.3	0.2	2.5	1.9	2.5	0.595	0.279	0.095
	**Lumbar**	4.9	0.7	4.8	3.1	7.2	4.1	0.9	3.6	1.7	7.5	5.4	1.2	3.7	1.2	13.0	8.2	1.5	8.0	5.2	11.7	0.092	0.458	0.186
**Plicometry (mm)**	**Chest ^○^**	10.6	1.2	9.5	8.0	15.0	10.3	2.2	8.3	5.0	19.0	8.0	0.7	7.3	5.5	13.0	12.9	2.1	14.0	7.0	16.5	0.996	0.255	0.061
	**Flank**	11.6	0.8	11.0	9.5	14.0	8.0	1.5	6.8	4.0	13.0	7.2	0.7	6.3	4.0	13.0	9.1	2.2	8.3	5.0	15.0	0.200	0.519	**0.037**
	**Lumbar**	12.2	1.3	12.0	8.5	16.0	8.4	1.0	7.9	5.5	12.5	8.7	0.5	8.3	6.0	12.0	10.0	1.3	8.8	8.5	14.0	0.338	0.215	**0.015**
	**Forechest**	13.0	1.2	14.0	8.5	15.0	9.1	1.9	8.5	4.5	14.0	7.3	0.8	6.5	3.5	12.0	8.1	1.9	7.8	4.0	13.0	**0.028**	0.297	0.106
	**Inguinal fold**	6.7	0.4	6.5	5.5	8.0	5.5	1.0	5.5	3.5	7.5	4.6	0.4	4.5	3.5	6.5	4.3	0.4	4.5	3.0	5.0	**0.011**	0.194	0.515

Hwi = height at withers, CC = chest circumference, PC = pelvic circumference, HS = hock to stifle length, OT = occipital-to-tail length, SC = skull circumference, SL = Skull length, TH = Tarsal pad–heel distance, CO = Carpal pad–olecranon distance, BF = body fat percentage, BMI = body mass index. ^†^ Analyzed using a non-parametric approach. ^○^ Data log-transformed prior to statistical analysis due to deviations from normality. Raw (untransformed) data are presented in the table for clarity. ^‡^ Calculated using sex-specific formulas.

**Table 3:** Crosstabulation of the body condition score (BCS) by sex and neuter status in sled dogs. Different letters indicate statistically significant differences in column proportions (*z*-test; *p* < 0.05).

		Sex and Neuter Status				Total
		Intact Female	Spayed Female	Intact Male	Neutered Male	
**BCS**	**4**	1 _a,b,c_ (20.0%)	0 _c_ (0.0%)	7 _b_ (58.3%)	0 _a,c_ (0.0%)	8 (29.6%)
	**5**	3 _a_ (60.0%)	3 _a_ (50.0%)	5 _a_ (41.7%)	0 _a_ (0.0%)	11 (40.7%)
	**6**	0 _a_ (0.0%)	1 _a_ (16.7%)	0 _a_ (0.0%)	4 _b_ (100.0%)	5 (18.5%)
	**7**	1 _a,b_ (20.0%)	2 _b_ (33.3%)	0 _a_ (0.0%)	0 _a,b_ (0.0%)	3 (11.1%)
**Total**		5 (100.0%)	6 (100.0%)	12 (100.0%)	4 (100.0%)	27 (100.0%)

**Table 4:** Intra- and inter-observer reliability of plicometry. Intraclass correlation coefficients (ICCs) of parameters evaluated using the plicometry assessed by two testers. Each ICC is followed by its 95% confidence interval (CI) and by the *p* value of the F test.

Parameter	Intra-Observer Agreement				Inter-Observer Agreement			
	ICC	95% Confidence Interval		*p* Value	ICC	95% Confidence Interval		*p* Value
		Lower Bound	Upper Bound			Lower Bound	Upper Bound	
**Chest**	0.782	0.557	0.897	<0.001	0.765	0.329	0.905	<0.001
**Flank**	0.614	0.311	0.804	<0.001	0.713	0.373	0.869	<0.001
**Lumbar**	0.695	0.440	0.848	<0.001	0.614	0.182	0.821	<0.001
**Forechest**	0.782	0.577	0.894	<0.001	0.339	−0.356	0.689	0.133
**Inguinal fold**	0.674	0.299	0.862	<0.001	0.270	−0.208	0.651	0.036

## Data Availability

The original contributions presented in this study are included in the article/Supplementary Materials.

## Supplementary Materials

The following supporting information can be downloaded at: https://www.mdpi.com/article/10.3390/vetsci12080766/s1, Table S1: Parameter estimates and goodness of fit of generalized linear models where the BCS was included as the dependent variable and objective measurements as independent.
