# Supplementary material for: Assessment of Body Condition in Long-Distance Sled Dogs: Validation of the Body Condition Score and Its Association with Ultrasonographic, Plicometric, and Anthropometric Measurements
Source: Vet Sci. 2025 Aug 16;12(8):766. doi: 10.3390/vetsci12080766 (PMC12390304; doi:10.3390/vetsci12080766)
Supplement: Supplementary file 1 [file vetsci-12-00766-s001.zip › vetsci-3798113-supplementary.pdf]

Supplementary material

Table S1. Parameter Estimates and goodness of fit of Generalised linear models where the BCS was included as the dependent variable and objective measurements as independent. P-values in bold are significant at the 0.05 level. OR= odds ratio; CI= confidence interval

Technique	Parameter	Parameter estimates			
		OR	95% CI for Exp(B)		Sig.
			Lower	Upper	
**Anthropometric measurements**	Body weight (kg)	1.164	0.941	1.439	0.162
	Height at withers (cm)	1.164	0.941	1.439	0.633
	Chest girth (cm)*	1.159	0.981	1.369	0.082
	Pelvic circumference (cm)	1.095	0.967	1.239	0.152
	Hock-to-stifle length (cm)	1.041	0.688	1.573	0.851
	Occipital-to-tail length (cm)	1.014	0.885	1.161	0.844
	Skull circumference (cm)	0.876	0.704	1.091	0.236
	Skull length (cm)	0.916	0.542	1.549	0.745
	Tarsal pad - heel distance (cm)	1.057	0.454	2.461	0.898
	Carpal pad - olecranon distance (cm)	0.985	0.751	1.292	0.914
**Calculated index**	Body Fat percentage (%BF)	1.067	.944	1.206	0.297
	Body Mass Index (BMI)*	1.284	1.062	1.553	**0.010**
**Measurements obtained with ultrasonography**	Chest	1.249	.669	2.333	0.485
	Flank	.982	.570	1.691	0.949
	Medial thigh	1.866	.412	8.458	0.418
	Lumbar	1.004	.982	1.026	0.723
**Measurements obtained with plicometry**	Chest*	1.521	1.194	1.939	**0.001**
	Flank*	1.347	1.061	1.710	**0.015**
	Forechest*	1.178	.974	1.424	0.092
	Inguinal fold *	2.134	.872	5.224	0.097
	Lumbar*	1.276	.980	1.660	0.070

*Variables with $p<0.1$ were included in the subsequent multivariate analyses.
